# Analysis of Chemical Composition, Antioxidant Activity, and Toxicity of Essential Oil from *Virola sebifera* Aubl (Myristicaceae)

**DOI:** 10.3390/molecules29143431

**Published:** 2024-07-22

**Authors:** Jorddy Neves Cruz, Mozaniel Santana de Oliveira, Oberdan Oliveira Ferreira, Antonio Rafael Quadros Gomes, Suraj N. Mali, Soluan Felipe Melo Pereira, Sabah Ansar, Cleydson Breno Rodrigues dos Santos, Rafael Rodrigues Lima, Eloisa Helena Aguiar de Andrade

**Affiliations:** 1 Adolpho Ducke Laboratory, Botany Coordination, Paraense Emílio Museum, Belém 66075-110, PA, Brazil; mozaniel.oliveira@yahoo.com.br (M.S.d.O.);; 2 Laboratory of Functional and Structural Biology, Institute of Biological Sciences, Federal University of Pará, Belém 66075-110, PA, Brazil; 3 Center for Biological and Health Sciences, State University of Pará, Tucurui 68600-000, PA, Brazil; rafaelquadros13@hotmail.com; 4 School of Pharmacy, D.Y. Patil University, Sector 7, Nerul, Navi Mumbai 400706, India; 5 Department of Clinical Laboratory Sciences, College of Applied Medical Sciences, King Saud University, P.O. Box 10219, Riyadh 11433, Saudi Arabia; sansar@ksu.edu.sa; 6 Laboratory of Modeling and Computational Chemistry, Department of Biological and Health Sciences, Federal University of Amapá, Macapá 68903-230, AP, Brazil; breno@unifap.br; 7 Faculty of Chemistry, Federal University of Pará, Belém 66075-110, PA, Brazil

**Keywords:** *Myristicaceae*, *Virola*, Amazon, essential oil, seasonal variation, antioxidant, toxicity

## Abstract

Volatile oils or essential oils (EOs) were extracted from three *V. sebifera* samples (labeled as A, B, and C) in September 2018 and February 2019; the extraction process involved hydrodistillation of the leaves. The chemical compositions of the EOs were analyzed using gas chromatography-mass spectrometry (GC/MS). The volatile components were identified by comparing their retention indices and mass spectra with standard substances documented in the literature (ADAMS). The antioxidant activity of the EOs was evaluated using 2, 2-diphenyl-1-picrylhydrazyl (DPPH), while their toxicity was assessed using *Artemia salina* Leach. Molecular docking was utilized to examine the interaction between the major constituents of *V. sebifera* EO and acetylcholinesterase (AChE), a molecular target linked to toxicity in *A. salina* models. The EO obtained from specimen A, collected in September 2018, was characterized by being primarily composed of (E,E)-α-farnesene (47.57%), (E)-caryophyllene (12.26%), and α-pinene (6.93%). Conversely, the EO from specimen A, collected in February 2019, was predominantly composed of (E,E)-α-farnesene (42.82%), (E)-caryophyllene (16.02%), and bicyclogermacrene (8.85%), the EO from specimen B, collected in September 2018, primarily contained (E,E)-α-farnesene (47.65%), (E)-caryophyllene (19.67%), and α-pinene (11.95%), and the EO from the leaves collected in February 2019 was characterized by (E,E)-α-farnesene (23.57%), (E)-caryophyllene (19.34%), and germacrene D (7.33%). The EO from the leaves collected in September 2018 contained (E,E)-α-farnesene (26.65%), (E)-caryophyllene (15.7%), and germacrene D (7.72%), while the EO from the leaves collected in February 2019 was primarily characterized by (E,E)-α-farnesene (37.43%), (E)-caryophyllene (21.4%), and α-pinene (16.91%). Among these EOs, sample B collected in February 2019 demonstrated the highest potential for inhibiting free radicals, with an inhibition rate of 34.74%. Conversely, the EOs from specimen A exhibited the highest toxic potentials, with an lethal concentration 50 (LC_50_) value of 57.62 ± 1.53 µg/mL, while specimen B had an LC_50_ value of 74.72 ± 2.86 µg/mL. Molecular docking results suggested that hydrophobic interactions significantly contributed to the binding of the major compounds in the EO from sample B to the binding pocket of AChE.

## 1. Introduction

*Virola sebifera*, a species from the Myristicaceae family, is a tree indigenous to the tropical regions of South and Central America. Often referred to as “Ucuuba,” it has a long-standing history of traditional medicinal use. The bark, leaves, and fruits of *V. sebifera* are traditionally employed in folk medicine due to their acknowledged anti-inflammatory, analgesic, and antispasmodic properties [1,2]. The plant boasts a diverse assortment of compound classes, including alkaloids, flavonoids, terpenes, and lignans, which are instrumental in producing its therapeutic effects [3,4]. In recent times, *V. sebifera* has gained prominence as a key ingredient in the cosmetic industry, primarily due to its moisturizing and nourishing attributes. Additionally, it is rich in essential oils (EOs), which are renowned for their medicinal properties and can be extracted from its bark, leaves, and fruits [5,6].

The EO derived from *V. sebifera* is notably rich in monoterpenes and sesquiterpenes. These compounds possess a range of beneficial properties, including anti-inflammatory, antioxidant, analgesic, antifungal, and antibacterial activities [7,8]. The oil is commonly obtained through steam distillation, as well as hydrodistillation from various parts of the plant, such as the bark, leaves, and other relevant plant components [9,10]. EOs are concentrated extracts derived from plants and are widely used in aromatherapy, massages, and other holistic therapies due to their natural properties. Each essential oil has a distinctive chemical composition, which directly contributes to its unique therapeutic potential [11,12]. For instance, the *Lavandula* spp. essential oil is recognized for its antifungal, antibacterial, smooth muscle relaxant, antidepressant, and sedative properties, and peppermint oil is commonly utilized for relieving symptoms of nausea and vomiting [13]. Depending on the specific oil and its intended usage, EOs can be consumed, breathed, or used topically. However, it is crucial to note that not all EOs are acceptable for internal intake, and consumption should only be carried out with the approval of a trained healthcare practitioner. Topical application and inhalation are more common and considered safe for most EOs when used appropriately [14]. Certain EOs are quite potent; thus, it is imperative to use caution and moderation when utilizing them. Some individuals may be more sensitive to certain EOs and experience skin irritation or allergic reactions. Before using the essential oil more broadly, it is advisable to conduct a patch test on a small patch of skin, as well as adhere to the recommended dilution guidelines; if any adverse reactions occur, it is advisable to discontinue use and seek medical advice if needed [15]. 

It is crucial to conduct studies on the toxicity of EOs. The brine shrimp lethality assay (BSLA) method is a widely used and cost-effective approach for evaluating the preliminary toxicity of various substances, including EOs. This assay provides valuable information regarding the potential toxicity of EOs and serves as a simple screening tool in early toxicity assessments [16]. The BSLA utilizes *A. salina* larvae, commonly known as brine shrimp, as a model organism. These larvae are known to be sensitive to a wide range of toxic substances, making them a suitable model for initial toxicity screening. The assay involves exposing the brine shrimp larvae to different concentrations of a substance, such as an essential oil, and observing their mortality rate. The results obtained from the BSLA can provide valuable insights into the potential toxicity of the tested substance [17]. The BSLA is conducted as follows. Brine shrimp larvae are hatched and exposed to a solution containing the substance under investigation. The larvae are then monitored for a designated period, often 24 h, and the number of deceased larvae is recorded. The toxicity of the substance is quantified as the lethal concentration 50 (LC_50_) value, which signifies the concentration at which 50% of the larvae perish. EOs have been found to exhibit varying levels of toxicity in the BSLA. The specific oil being tested, the concentration used, and the length of exposure to the larvae are some of the variables that can affect an essential oil’s toxicity in the BSLA. By assessing these factors, the BSLA offers valuable insights into the toxicity profiles of EOs [18,19,20]. Notably, although BSLA is a useful tool for initial toxicity screening, it should not be considered a substitute for comprehensive toxicity testing in animal models and humans; while it offers preliminary data regarding a substance’s potential toxicity, it is not always accurate in forecasting the toxicity levels and outcomes in more complex species. To fully assess the safety and toxicity of EOs, additional research with suitable animal models and, eventually, clinical trials including humans are required. These studies provide a more precise understanding of the safety profile and potential dangers related to the use of EOs by considering variables including dosage, systemic effects, and potential interactions [21,22].

The molecular modeling of *A. salina* AChE employs computational techniques to investigate its 3D structure and function at the molecular level [23]. AChE is a crucial enzyme involved in the regulation of the neurotransmitter acetylcholine in the nervous system of *A. salina* and other organisms [24,25]. The design of new medications or inhibitors targeting AChE can benefit from this molecular modeling, which illuminates the enzyme’s mechanism of action. These simulations offer insights into the protein structure’s dynamics and stability, as well as the interactions between AChE and its ligands [26,27]. Molecular modeling of AChE has been instrumental in designing new drugs for the treatment of Alzheimer’s disease, a condition characterized by a decrease in brain acetylcholine levels [28]. Furthermore, molecular modeling has been utilized to examine the impact of environmental toxins on AChE activity in *A. salina*, providing insights into these compounds’ toxicity mechanisms [29]. In this context, this study aims to reveal new information about the chemical composition of *V. sebifera* EOs and to assess its biological potential and molecular interaction mechanism with AChE.

## 2. Results

### 2.1. Yield of 2 EOs

Table 1 lists the yield of EOs (mL/80 g) extracted from the leaves of three *V. sebifera* specimens (A, B, and C), collected in September 2018 and February 2019, as well as their respective moisture contents (%), illustrating the variations in the essential oil contents across the specimens.

The highest oil production was recorded in February, with values of 0.8, 0.7, and 0.69 for specimens A, B, and C, respectively. Specimen A consistently demonstrated the highest content during both collection periods. The yield obtained during our study typically surpassed yields from the EOs of *V. calophylla*, *V. multinervia*, and *V. pavonis*, which exhibited contents of 0.19%, 0.10%, and 0.12%, respectively [30].

### 2.2. Chemical Composition

Table 2 lists the 72 chemical constituents identified in the EOs derived from the leaves of the three *V. sebifera* specimens. These specimens were collected in September 2018 during the dry season, and in February 2019 during the rainy season.

The hydrocarbon monoterpene class constitutes a moderate percentage of the EO composition. A notable quantitative variation is observed in this compound class when comparing the percentages of these substances among different specimens collected during the same period. However, no significant variation is detected in other compound classes. For the EOs procured in September 2018, the percentage difference in the hydrocarbon monoterpene class is relatively minor; specimens A, B, and C demonstrate percentages of 9.64%, 14.72%, and 8.5%, respectively. In contrast, the oils collected in February 2019 show a substantial difference in these compounds’ quantities; specimens A and B exhibit percentages of 3.37% and 4.58%, respectively, while specimen C presents a significantly higher percentage of 18.75%, marking a difference of 15.38% and 14.17% compared to specimens A and B, respectively. Furthermore, there is a notable variation in the hydrocarbon monoterpene compounds’ content among the different EOs.

In this study, the class of oxygenated monoterpenes is the least represented in the four essential oil samples obtained from the evaluated specimens. The essential oil from specimen A contained 0.49% and 0.68% of oxygenated monoterpenes in September 2018 and February 2019, respectively. Specimen B exhibited 0.29% of these compounds in September 2018, and in February 2019, the oxygenated monoterpenes showed the lowest value among all the essential oil samples, with a mere 0.25% of these substances detected.

Sesquiterpene hydrocarbons were the predominant class of compounds in all three specimens evaluated across both collection periods. In September 2018, these compounds constituted 84.63% of the EO in specimen A, increasing to 89.04% in February 2019, the highest recorded percentage of sesquiterpene hydrocarbons across all samples. In specimen B, the sesquiterpene hydrocarbons made up 81.79% and 84.97% of the EO in September 2018 and February 2019, respectively. Meanwhile, specimen C contained 79.81% sesquiterpene hydrocarbons in September 2018, decreasing to 74.01% in February 2019.

Oxidized sesquiterpenes constituted 4.41% and 4.98% of the EO in specimen A in the September 2018 and February 2019 collections, respectively. In contrast, specimen B’s September 2018 collection exhibited the lowest concentration of this compound class among the evaluated EOs, comprising only 1.65% of the total. However, by February 2019, the concentration had risen to 5.71%, attributable to the emergence of substances not previously identified in the September 2018 essential oil. The September 2018 EO collection from specimen C revealed the highest oxidized sesquiterpenes concentration; during this period, these compounds accounted for 7.28% of the oil’s molecular composition, while in February 2019, the same specimen contained 3.28% of these compounds.

**Figure 1:**
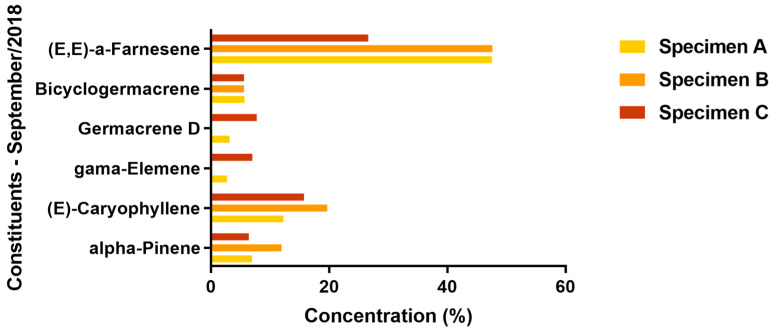
Variation of the major constituents of the three specimens of *V. sebifera* collected in September 2018 (Amazonian summer).

**Figure 2:**
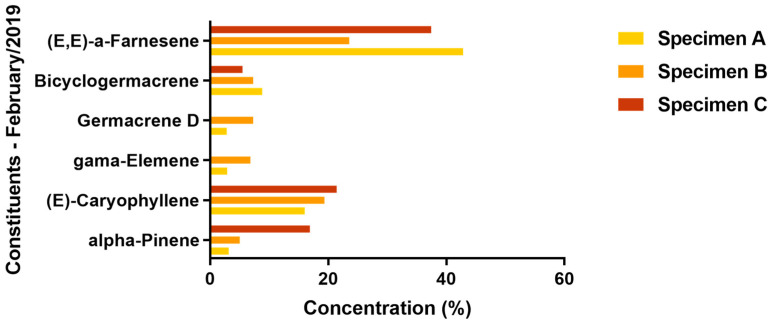
Variation of the major constituents of the three specimens of *V. sebifera* collected in February 2019 (Amazonian winter).

Figure 1 and Figure 2 present the variations of the major chemical constituents (≥5%) identified in the leaves of the three specimens.

The transition from the dry to the rainy season in the Amazon region generally influences the proportion of major constituents in the specimens. It is crucial to highlight that the Amazon’s climatic shifts are marked by unique weather patterns. The dry season, characterized by the hottest months of the year, sees temperatures reaching approximately 38 ºC; conversely, the rainy season is marked by frequent precipitation, the possibility of cloud cover, and persistently high temperatures [31,32,33].

During both collection periods, the sesquiterpene hydrocarbon (E,E)-α-farnesene was the predominant component in the oil samples from all three specimens, with its concentration ranging from a low of 23.57% to a high of 47.65%; both of these extremes were observed in specimen B, during the Amazonian summer and winter periods, respectively. Specimens A and B were particularly notable for their production of this compound during the Amazonian summer, with it constituting 47.57% and 47.65% of their respective oil compositions; however, it is important to note that specimen C also produced a significant amount of this compound during the same period, with it making up 26.65% of the essential oil.

The shift from summer to the rainy season in the Amazon region influenced the production of α-pinene, particularly in specimen C. This seasonal change resulted in a reduction in this compound in specimens A and B; conversely, specimen C demonstrated an increase in α-pinene production, with percentages rising from 6.37% in September 2018 to 16.91% in February 2019.

In general, the percentage of (E)-caryophyllene in the oil of the three specimens increased during the transition from the Amazonian summer to winter. Only a very small decrease (0.33%) in the quantity of this compound was observed in specimen B.

The EO of specimen B collected in September 2018 did not exhibit the presence of γ-elemene. Similarly, this compound was absent in the EO of specimen C collected in February 2019, during the Amazonian winter. However, specimen A consistently produced a similar percentage of this compound across both evaluated periods. These findings suggest that the regional rainfall index may influence the production of γ-elemene. Although other factors could potentially exist, our analysis primarily highlights this climatic influence due to the varying collection times of the plant material throughout the year. A comparable trend was observed for germacrene D, which was absent in the EOs from both specimen B in September 2018 and specimen C in February 2019.

In the transition periods from summer to winter, specifically in September 2018 and February 2019, the production of bicyclogermacrene saw an increase corresponding to the times when the plant material was harvested.

The EOs examined in our study exhibited a chemical profile distinct from those of *V. calophylla*, *V. multinervia*, and *V. pavonis*. The essential oil of *V. calophylla* is primarily composed of β-caryophyllene (54.8%), bicyclogermacrene (10.0%), and α-humulene (8.6%); the essential oil of *V. multinervia* was characterized by a predominance of β-caryophyllene (55.7%), caryophyllene oxide (9.8%), and α-humulene (5.0%); and the essential oil of *V. pavonis* was characterized by the major compounds β-selinene (60.5%), β-caryophyllene (12.7%), and β-Elemene (4.1%) [30].

Farnesene derivatives, including the primary component of the current sample, are documented in the literature to possess biological properties, such as modulatory effects on human neutrophil inflammation and potential antifungal activity against fungi prevalent in apple trees [34,35]. (E)-caryophyllene demonstrates potential properties such as anticancer, analgesic, repellent, and anti-inflammatory, among others [36,37]. α-pinene is known for its antimicrobial, cytotoxic, and antitumor activities [38,39,40]. Bicyclogermacrene is associated with larvicidal activity and antiviral activity against SARS-CoV-2 [41]. Germacrene D is recognized for its anti-inflammatory, cytotoxic, analgesic, and anticancer properties [42,43]. γ-elemene is noted for its larvicidal, antibacterial, and antitumor activities [44,45,46].

Da Silva et al. [47] noted an increase in the secondary metabolism of aromatic plants, specifically in the production of the sesquiterpenes germacrene D, bicyclogermacrene, and elemene derivatives, during rainy periods. They note that this concentration increase may be a strategic response to attract a new guild of pollinators, such as bees and flies, which are more prevalent during these periods. Similarly, Cascaes et al. [48] suggested that the Amazon region’s rainy season, characterized by higher relative humidity and lower solar radiation, results in elevated levels of (E)-caryophyllene, germacrene D, elemene derivatives, and bicyclogermacrene.

### 2.3. Antioxidant Capacity Equivalent to Trolox of V. sibifera Essential Oil

**Figure 3:**
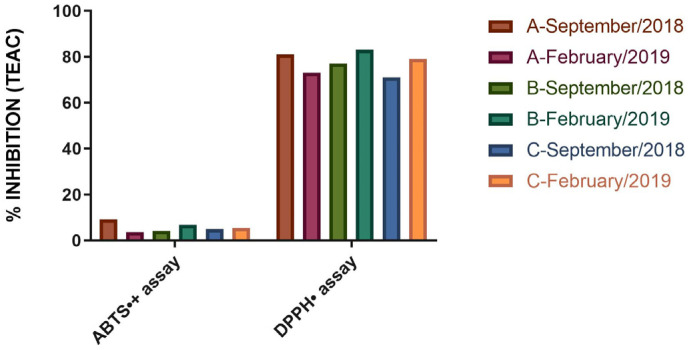
% Inhibition of Antioxidant capacity equivalent to Trolox by scavenging ABTS•+ and DPPH• from *V. sebifera.* Values are expressed as mean and standard deviation (n = 3) of Trolox equivalent antioxidant capacity by scavenging ABTS•+ and DPPH•.

The Trolox equivalent antioxidant capacity (TEAC) by 2, 2-diphenyl-1-picrylhydrazyl (DPPH) scavenging of the EO from specimen A collected in September 2018 and February 2019 was found to be 0.81 ± 0.05 (81.0% inhibition) and 0.73 ± 0.07 Mm (73.0% inhibition), respectively; in the same period, specimen B exhibited values of 0.77 ± 0.04 (77.0% inhibition) and 0.83 ± 0.06 (83.0% inhibition) mM. As for the EO samples from specimen C, the values were 0.71 ± 0.08 (71.0% inhibition) and 0.79 ± 0.09 (79.0% inhibition) mM for the September 2018 and February 2019 collections, respectively (Figure 3).

In the ABTS•+ assay, the TEAC values for specimen A were recorded as 0.179 ± 0.005 (9.2% inhibition) and 0.065 ± 0.007 (3.6% inhibition) mM for the collections in September 2018 and February 2019, respectively. For specimen B, the recorded values were 0.082 ± 0.013 (4.16% inhibition) mM for September 2018 and 0.128 ± 0.007 (6.75% inhibition) mM for February 2019. Meanwhile, the TEAC values for specimen C were 0.093 ± 0.006 (4.95% inhibition) and 0.107 ± 0.004 (5.55% inhibition) mM for September 2018 and February 2019. The results demonstrated that the % inhibition of the DPPH radical was higher in relation to the ABTS radical assay, indicating that the DPPH radical quantification assay is more effective for evaluating the antioxidant activity of the oil. The difference in antioxidant activities between the techniques used is due to the ability of the DPPH technique to be more sensitive and specific in the abstraction of hydrogen atoms from phenolic groups in phenols or amino groups from aromatic amines, compared to TEAC. In other words, the difference in antioxidant quantification is related to the techniques and not to the solubility of the samples in water, with the DPPH technique being more efficient for detecting antioxidant compounds in this plant.

The literature offers scant TEAC results for the essential oil (EO) derived from *V. sebifera* specimens are scarce in the literature. However, Zhi et al. [49] and Zhao et al. [50] suggest that the major components of *V. sebifera*, such as (E,E)-α-farnesene and other farnesene derivatives, could potentially enhance its antioxidant capacity. Stanojevic et al. [51] reported that the EO of *Matricaria chamomilla* flowers primarily consists of farnesene derivatives (39.10%); they further noted that this EO demonstrates superior antioxidant properties following a 90-min incubation period, with an EC_50_ value of 2.07 mg/mL.

(E)-caryophyllene, a significant component in the EO of *V. sebifera*, is known for its ability to combat oxidative stress and mitochondrial dysfunction in the nervous system [52]. Dahham et al. [53] reported that this sesquiterpene demonstrates a TEAC by scavenging DPPH• equivalent to 1.25 ± 0.06 µM. In the FRAP assay, the antioxidant capacity was reported to be 3.23 ± 0.07 µM. These results, as per the authors, were marginally lower than those of ascorbic acid, which exhibited a TEAC of 1.50 ± 0.03 µM and 3.80 ± 0.04 µM for the DPPH• and FRAP assays, respectively.

Casiglia et al. [54] reported that the EO derived from the aerial parts of *Kundmannia sicula* primarily contains germacrene D (81.20%). The authors noted that this oil demonstrates significant scavenging activity against the ABTS•+ radical, with IC_50_ values of 14.5 μg/mL. Similarly, Franco et al. [41] reported that the EO extracted from the leaves of a *Myrcia tomentosa* specimen in the Amazon exhibited a comparable chemical composition to the specimens analyzed, with γ-elemene (12.52%), germacrene D (11.45%), and (E)-caryophyllene (10.22%) as the predominant components. They emphasized that this EO inhibits 53.60 ± 0.15% of ABTS•+ radicals and 213.00 ± 0.91% of DPPH• radicals.

Giorgi et al. [55] demonstrated that the hexane, ethanolic, and ethanolic-aqueous extracts from *Bixa orellana* seeds contain high concentrations of germacrene D, γ-elemene, and (E)-caryophyllene. These extracts, according to the authors, are excellent sources of antioxidant compounds with potential for DPPH• radical inhibition. Using in vitro analysis, Shahriari et al. [56] reported that α-pinene exhibits the most effective inhibition of the AChE enzyme, with an IC_50_ value of 0.8640 µL/mL; this was in contrast to trans-anethole and thymol, which had IC_50_ values equivalent to 0.4900 and 0.1370 µL/mL, respectively.

Jeribi et al. [57] identified elevated concentrations of bicyclogermacrene in the EO derived from the leaves, fruits, and seeds of *Schinus terebinthifolius*. These levels varied between 23.56% and 35.58%. The authors noted that the EO extracted from the fruits, despite having a lower bicyclogermacrene content, exhibited a higher total phenolic content, leading to increased antioxidant activity.

Generally, sesquiterpenes exhibit a lower antioxidant capacity than monoterpenes and phenylpropanoids [58]. Furthermore, organic compounds with conjugated carbon double bonds and/or hydroxyl groups tend to donate hydrogen to free radicals; this action stabilizes the radicals and mitigates the effects of lipid oxidation, a process responsible for the aggravation of diseases associated with oxidative stress, such as Alzheimer’s disease, Parkinson’s disease, and sclerosis, among others. Notable oxygenated compounds and/or those with a double bond that exhibit potential free radical inhibition activity include linalool, thymol, terpinen-4-ol, eucalyptol, limonene, terpilene, and pinene derivatives [59,60,61,62,63,64,65,66].

### 2.4. Toxicity Bioassay in Artemia salina

The calculation of the larval mortality rate was performed 24 h post-test. LC_50_ values were ascertained by transforming the larval mortality percentage into probits. The EOs tested demonstrated mortality rates between 51.17% and 70.92%, contingent on the concentration range. Table 3 displays the estimated mean LC_50_ values, accompanied by their respective coefficients of determination.

The microcrustacean *A. salina* is utilized in bioassays to screen for potentially toxic substances. There are instances where toxicity towards *A. salina* aligns with toxicity towards mammalian cells. As per the classification proposed by Ramos et al., essential oil is deemed toxic if it displays an LC_50_ below 80 μg/mL [67], moderately toxic if it ranges within 80–250 μg/mL, and non-toxic or slightly toxic if it exceeds 250 μg/mL.

The bioassay results reveal a high toxicity level in the EOs derived from both specimen A and specimen B of *V. sebifera* towards *A. salina*, with an LC_50_ equivalent to 70.92 ± 2.22 and 72.17 ± 5.43 μg/mL, respectively. Conversely, the essential oil from specimen C demonstrated a lower toxicity level towards the microcrustaceans, with an LC_50_ of 51.17 ± 3.95 μg/mL.

No literature reports exist on the preliminary toxicity of the essential oil from *V. sebifera* and other species of the same genus towards *A. salina*. However, Anunciação et al. [68] proposed that the EOs from the bark and leaves of *V. surinamensis* can be used as potential phytopharmaceuticals in treating uterine colon cancer due to their toxicological effects on this cancer type’s tumor cell lines. The authors further indicated that the essential oil from the leaves primarily consists of sesquiterpene hydrocarbons such as α-farnesene (14.50%), β-elemene (9.61%), and bicyclogermacrene (8.10%); conversely, the essential oil from the branches predominantly contains aristolene (28.40%), α-gurjenene (15.00%), and valencene (14.10%).

The toxicity of major components, particularly (E,E)-α-farnesene, has been minimally reported. Satyal et al. [69] and Machado et al. [70] noted potential toxic effects. They found that EOs from the aerial parts of *Matricaria chamomilla* and the leaves of *Lantana camara*, which contain farnesene and (E)-caryophyllene derivatives as primary components, display significant toxicity against *A. salina*, with LC_50_ values of 31.70 and 10.00 µg/mL, respectively. Additionally, Machado et al. [70] demonstrated the toxic effects of farnesene derivatives against *Leishmania* species.

Machado et al. [70] have reported that (E)-caryophyllene, a sesquiterpene, does not exhibit any toxic effects against *A. salina* over a 24-h exposure period; Francomano et al. [71] stated that this compound only demonstrates toxicity in humans when administered in high concentrations. Mitić et al. [19] found that the EOs derived from the branches of three *Abies* genus species primarily contain α-pinene and its isomer β-cinene as major volatile components. The authors have also noted that all EOs display high toxicity against *A. salina*, with an LC_50_ value of less than 80 µg/mL.

Paudel et al. [72] identified the primary components of *J. regia* and *J. nigra* EOs as farnesene derivatives, (E)-caryophyllene, and germacrene D. These EOs demonstrated toxicity against *A. salina*, with LC_50_ values under 80 µg/mL. Similarly, Judžentienė and Jurga Būdienė [73] found that the essential oil of *Artemisia vulgaris* primarily consists of germacrene D (10.60–30.50%); they reported that *Artemisia* EOs display significant toxicity in the bioassay with *A. salina*, with LC_50_ values between 10.25 and 19.11 µg/mL, revealing that EOs with substantial amounts of germacrene D are notably more toxic.

Benelli et al. [44] reported that γ-elemene and (E,E)-α-farnesene demonstrate significant larvicidal toxicity against the fourth instar larvae of *Spodoptera litura*, with LC_50_ values of 10.64 and 16.77 µg mL^−1^, respectively. Govindarajan et al. [45] found that biclogermacrene is toxic to mosquitoes of the *Anopheles subpictus*, *Anopheles albopictus*, and *Culex tritaeniorhynchus* species.

The analyzed EO activity against *A. salina* can be attributed to the presence of major components, as well as the synergistic or antagonistic effects produced by all sample components [36,74]. Generally, sesquiterpenes display greater toxicity than monoterpenes and phenylpropanoids [75]. These effects may contribute to the toxic effects on *A. salina* demonstrated by the essential oil from the three *V. sebifera* specimens.

The enzyme AChE is recognized as a target enzyme in aquatic organisms, including *A. salina*. Natural products are extensively researched for their potential as AChE inhibitors, given their potential utility in treating various neurological disorders [76,77,78]. Arslan et al. [79] state that farnesene derivatives show considerable anti-AChE activity when combined with β-amyloids, peptides used in the treatment of Alzheimer’s disease, exhibiting inhibitory activity equivalent to 474.80 µm L^−1^ after 24 h of exposure and 413.80 µm L^−1^ after 48 h. The authors further proposed that the sesquiterpene and its derivatives may serve as potentially safe, anti-necrotic, and neuroprotective agents against Alzheimer’s disease.

The literature reports that (E)-caryophyllene exhibits anti-AChE activity [80,81]. This sesquiterpene also synergistically interacts with phytocannabinoids, significantly enhancing its efficacy against AChE [82]. Formagio et al. [56] state that the essential oil of *Psychotria poeppigiana* contains germacrene D (29.38%) and bicyclogermacrene (25.21%) as major components; they claimed that this natural substance exhibits anti-AChE activity and anti-hyperalgesic properties, and thus can be used to treat pain and inflammation.

The monoterpenoid α-pinene demonstrates activity against the AChE enzyme found in bovine erythrocytes [83]. Calva et al. [84] reported that the essential oil derived from the aerial parts of *Myrteola phylicoides* contains α-pinene (30.94%), (E)-caryophyllene (21.93%), and β-pinene (14.45%) as its primary constituents and inhibits AChE at a concentration of 60.80 µg mL^−1^; they also emphasized that the main component of the essential oil does not contribute significantly to its anti-AChE activities and that further research is required to assess the interaction between the components present in this essential oil and AChE.

The sesquiterpene γ-elemene, a major component of the essential oil derived from various *Piper* species in the Brazilian Amazon, was reported by Da Silva [85]. The author noted that several *Piper* EOs display anti-AChE activity due to the presence of γ-elemene and other elemene-derived components, which inhibit proliferation, stimulate apoptosis, and induce cell cycle arrest in malignant cells. γ-elemene is among the antineoplastic sesquiterpenes found in numerous medicinal plants [86]. Hussein et al. [87] reported that the compound was docked with AChE with a binding affinity equivalent to −8.8 kcal mol^−1^ and subsequently exhibited hydrophobic interactions with acidic amino acid residues in the catalytic triad (His439, Trp83, Phe329, and Tyr333) and PAS (Asp71, Trp83, Ser80, Tyr120, Phe329, and Tyr333).

### 2.5. Molecular Docking

**Figure 4:**
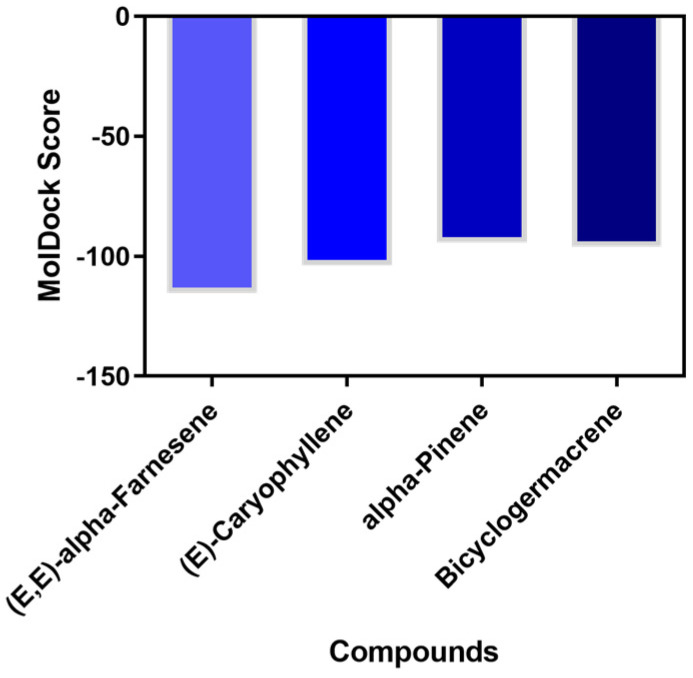
MolDock Score values of the evaluated complexes.

Various molecular modeling techniques are utilized to decipher the molecular interactions that facilitate the formation of complexes between natural compounds and biomolecules [88,89]. We used molecular docking to evaluate the interaction between the primary compounds of *V. sebifera* essential oil and the binding pocket of AChE. The MolDock Scores for the complexes formed by AChE with the ligands (E,E)-α-farnesene, (E)-caryophyllene, α-pinene, and bicyclogermacrene were −115.36, −103.68, −94.35, and −96.08, respectively (Figure 4).

**Figure 5:**
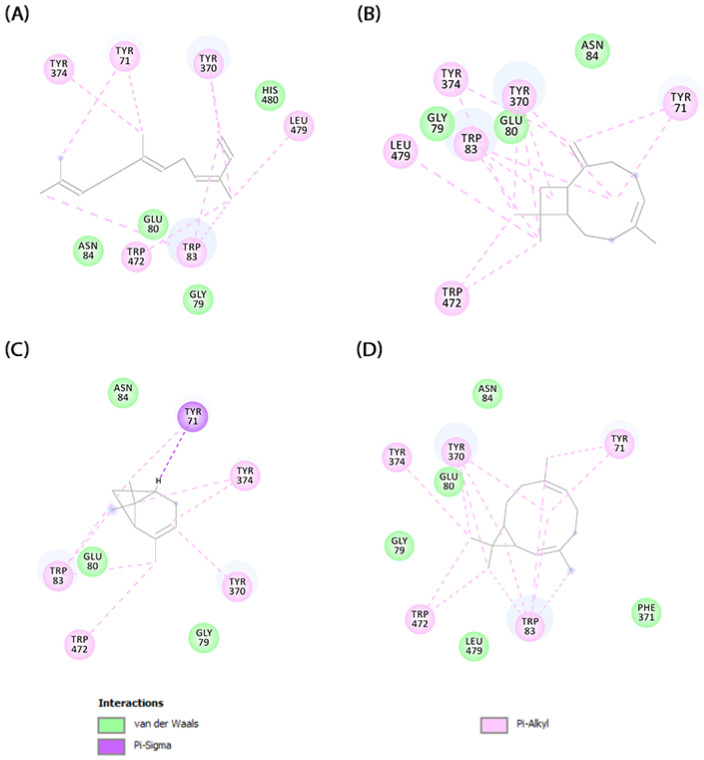
Interaction conformations obtained by molecular docking for the compounds (**A**) (E,E)-α-farnesene, (**B**) (E)-caryophyllene, (**C**) α-pinene, and (**D**) bicyclogermacrene.

The results indicate a spontaneous formation of the ligand-AChE complex. Following the complex formation, we assessed the molecular binding mode and the chemical interactions that sustain the compounds’ interaction with the enzyme’s active site (Figure 5).

The primary factor directing ligand interaction within the protein binding cavity is hydrophobic interactions. (E,E)-α-farnesene establishes pi-alkyl interactions with TYR374, TYR71, TYR370, LEU479, TRP472, and TRP83, whereas van der Waals interactions are established with ASN84, GLU80, and GLY79. (E)-caryophyllene create multiple pi-alkyl hydrophobic interactions with LEU479, TYR374, TYR370, TRP83, TYR71, and TRP472, along with three van der Waals interactions with GLY79, GLU80, and ASN84. α-pinene demonstrates a pi-sigma interaction with TYR71 and pi-alkyl interactions with TRP472, TRP83, TYR374, and TYR370, as well as van der Waals interactions with GLU80, ASN84, and GLY79. Bicyclogermacrene establishes van der Waals interactions with GLY79, GLU80, ASN84, LEU479, and PHE371, and pi-alkyl hydrophobic interactions with TRP472, TYR374, TYR370, TYR71, and TRP83 within the enzyme’s binding pocket.

### 2.6. Multivariate Analysis

**Figure 6:**
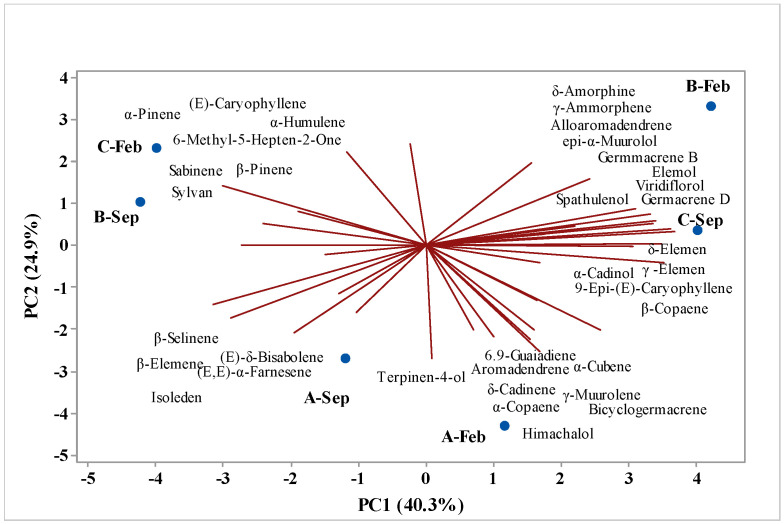
PCA analysis of compounds identified in EOs from *V. sebifera*.

**Figure 7:**
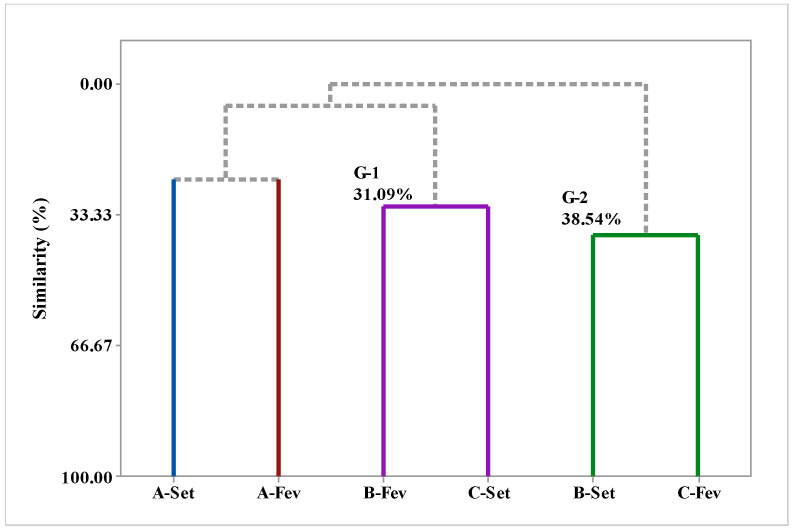
Similarity relationship of the compounds identified in EOs from *V. sebifera*.

Principal component analysis (PCA) (Figure 1) and hierarchical cluster analysis (HCA) were utilized to examine the correlation between the chemical compounds identified in various fractions of EOs extracted from *V. sebifera* during different seasons of the year, specifically the dry and rainy periods; these correlations are depicted in Figure 6 and Figure 7, respectively. Figure 5 illustrates the main analyzed components, PC1 and PC2; PC1 accounts for 40.3% of the variables, while PC2 accounts for 24.9%, together accounting for a total of 65.2% of the variance in the analyzed data.

The HCA analysis assesses the similarity among the identified compounds. Two distinct groups are formed, with two samples, A-Sep and A-Feb, lacking sufficient similarity to form a group. The EOs isolated from *Virola* leaves collected in the months of B-Feb and C-Sep form a group with a similarity level of 31.09%, whereas samples B-Sep and B-Feb exhibit the highest similarity level of 38.54%. Upon examining Figure 7, we can observe the substances with the highest weights, i.e., those that contribute to the formation of groups. A-Sep is characterized by the presence of β-selinene, β-elemene, (E)-δ-bisabolene, (E,E)-α-farnesene, and isoleden. By contrast, sample A-Feb is characterized by the presence of 6,9-guaiadiene, aromadrene, α-cubene, δ-cadinene, α-copaene, himacholol, γ-muurolene, and bicyclogermacrene. The group comprising samples B-Feb and C-Sep is characterized by the compounds (E)-caryophyllene, α-pinene, 6-methyl-5-hepten-2-one, sabinene, β-pinene, and sylvan; conversely, the group consisting of samples B-Sep and B-Feb is characterized by δ-amorphene, γ-amorphene, alloaromadrene, epi-α-muurolol, germacrene B, elemol, viridiflorol, germacrene D, spathulenol, δ-elemene, γ-elemene, α-cadinol, 9-epi-(E)-caryophyllene, and β-copaene.

## 3. Materials and Methods

### 3.1. Botanical Material

The botanical material was collected from three specimens of *V. sebifora* grown in the research campus of the Emilio Goeldi Museum (MPEG) in Belém, Pará. Each specimen was given an identification: samples A, B, and C. The collected plant specimens were preserved and added to the collection of the João Murça Pires Herbarium (MG) at the Emílio Goeldi Museum. They were specifically included in the Aromatic Plants of the Amazon collection in Belém, Pará. The specimens were assigned the following registration numbers: MG236908 (*V. sebifora*, specimen A), MG210943 (*V. sebifora*, specimen B), MG1621198 (*V. sebifora*, specimen C).

### 3.2. Preparation and Characterization of Botanical Material

The leaf samples from the specimens were dried in an air-circulating oven at a temperature of 35 °C for 5 days. Subsequently, they were ground using a knife mill (Tecnal, model TE-631/3, Piracicaba, Brazil), homogenized, weighed, and subjected to the extraction process. The moisture content was analyzed using an infrared moisture analyzer (ID50; GEHAKA, Duquesa de Góias, Real Parque, São Paulo—Brazil).

### 3.3. Extraction of EOs

The samples were hydro-distilled for 3 h in modified Clevenger-type glass systems with a cooling mechanism to keep the condensation water at about 12 °C. Following the extraction, the oils were centrifuged for 5 min at 3000 rpm, dehydrated with anhydrous sodium sulfate, and then centrifuged once more under the same circumstances. The oil yield was calculated as mL/100 g. The oils were kept in flame-sealed amber glass ampoules and kept at 5 °C in a refrigerator.

### 3.4. Calculation of Yield (%) of Extracted Oil

The yield (%) of the extracted essential oil was obtained from the dried and moisture-free crude material (DMC).

The gross oil yield was calculated by relating the volume of the obtained oil to the mass of the plant material used.

(1) $$\mathrm{Gross}\;\mathrm{oil}\;\mathrm{yield}=\frac{volume\;of\;obtained\;oil\;\left(mL\right)}{mass\;of\;plant\;material\;\left(g\right)}\times 100$$

The calculation of the yield of oil on a moisture-free basis (MFY) was executed- by relating the mass of oil to the moisture content as follows:

(2) $$oilBLU=\frac{volume\;of\;oil\;obtained\left(mL\right)}{m\left(g\right)-\left(\frac{m\left(g\right)\times U}{100}\right)}\times 100$$

### 3.5. Chemical Composition Analysis

The chemical compositions of the EOs of *V. sebifora* (specimen A, B and C), were analyzed using a Shimadzu QP-2010 plus (Kyoto, Japan) a gas chromatography system equipped with an Rtx-5MS capillary column (30 m × 0.25 mm; 0.25 µm film thickness) (Restek Corporation, Bellefonte, PA, USA) coupled to a mass spectrometer (GC/MS) (Shimadzu, Kyoto, Japan). The programmed temperature was maintained at 60–240 °C at a rate of 3 °C/min, with an injector temperature of 250 °C, helium as the carrier gas (linear velocity of 32 cm/s, measured at 100 °C), and a splitless injection (1 μL of a 2:1000 hexane solution), using the same operating conditions as described in the literature [90]. The components were quantified using gas chromatography (GC) on a Shimadzu QP-2010 system (Kyoto, Japan) equipped with a flame ionization detector (FID) (Kyoto, Japan), under the same operating conditions as before, except for the carrier hydrogen gas. The retention index for all volatile constituents was calculated using a homologous series of *n*-alkanes (C8–C40) Sigma-Aldrich (San Luis, MO, USA), according to Van den Dool and Kratz [91]. The components were identified by comparison (i) with that of the experimental mass spectra with those compiled in libraries (reference) and (ii) with their retention indices to those found in the literature [92,93].

### 3.6. Trolox Equivalent Antioxidant Capacity (TEAC)

The 2,2’-Azino-bis (3-ethylbenzothiazoline-6-sulfonic acid (ABTS•+) and 2,2-diphenyl-1-picrylhydrazyl (DPPH•) radicals scavenging assays were used for the assessment of the antioxidant capacity of EO, determined according to their equivalence to the potent antioxidant, Trolox (6-hydroxy-2,5,7,8-tetramethylchromono-2-carboxylic acid; Sigma-Aldrich; 23881-3; São Paulo, Brazil), and a water-soluble synthetic vitamin E analog.

#### 3.6.1. ABTS•+ Assay

The ABTS•+ radical scavenging assay was determined according to the methodology adapted from Miller et al., [94] and modified by Re et al., [95]. ABTS•+ (Sigma-Aldrich; A1888; São Paulo, Brazil) was prepared using 7 mM ABTS and 140 mM of potassium persulfate (K_2_O_8_S_2_; Sigma Aldrich; 216224; São Paulo, Brazil) incubated at room temperature without light for 16 h. Then, the solution was diluted with phosphate-buffered saline until it reached an absorbance of 0.700 ± 0.02 at 734 nm.

To measure the antioxidant capacity, 2.97 mL of the ABTS•+ solution was transferred to the cuvette, and the absorbance at 734 nm was determined using a Biospectro SP 22 spectrophotometer (São Paulo, Brazil). Then, 0.03 mL of the sample was added to the cuvette containing the ABTS•+ radical, and after 5 min, the second reading was performed. The Trolox was used as a standard solution for the calibration curve (y = 0.455x + 0.0002), where y represents the value of absorbance and x is the value of concentration, expressed as mM; R^2^ = 0.998).

#### 3.6.2. DPPH• Radical Scavenging Assay

The test was carried out according to the method proposed by [96]. To measure the antioxidant capacity, initially, the absorbance of DPPH• solution (Sigma-Aldrich; D9132; São Paulo, Brazil) 0.1 mM diluted in ethanol was determined. Subsequently, 0.6 mL of DPPH• solution, 0.35 mL of distilled water, and 0.05 mL of the sample were mixed and placed in a water bath at 30 °C for 30 min. Thereafter, the absorbance was determined using a spectrophotometer Bioespectro SP 22 (São Paulo, Brazil) at 517 nm. The synthetic antioxidant Trolox was used as a standard solution for the calibration curve (y = 0.2261x − 0.0094), where y represents the value of absorbance and x is the value of concentration, expressed as mM; R^2^ = 0.9831).

### 3.7. Preliminary Toxicity Bioassay with Artemia salina Leach

For the toxicity tests, the sample with the highest mass yield was selected. The preliminary toxicity bioassay test of *V. sebifora* (specimen A, B and C) essential oil in *A. salina* Leach was performed as described in the literature [29,97]. The essential oil was prepared at concentrations of 100, 50, 20, 10, 5, and 1 μg mL−1 were used, from fresh and dry samples obtained in the seasonal study in the months of July, September, November, January, March, and May. A total of ten *A. salina* larvae were added to each test flask with the aid of automatic micropipettes. Brine water (artificial) and DMSO were used as solvents with a 95:5 ratio. In the control group and the positive group with lapachol, the same solvent was used for the samples and larvae under the same conditions as the bioassay. After 24 h of contact between the larvae and the sample solution, the dead larvae were counted (in each concentration), and the mortality rate and the IC_50_ value were calculated using the Probitos statistical method. All the experiments were performed in triplicate (n = 3).

### 3.8. In Silico Evaluation of MAJOR EO Components

We utilized molecular docking to assess how the major compounds of *V. sebifera* EO (specimen A—collected in September) interact with the binding pocket of AChE. This molecular target was selected due to its association with toxicity mechanisms in *A. salina*. The major compounds of this EO were used as models for interaction, as they exhibited higher toxicity in *A. salina* models. The three-dimensional structure of the AChE protein was obtained from the Protein Data Bank with the ID 1QON [98]. The substances used in our studies were retrieved from PubChem, and the molecular structures of these compounds were optimized using the B3LYP/6-31G method [99,100] with Gaussian 09 [101]. To evaluate the molecular binding mode, we employed Molegro Virtual Docker 5.5 software [102]. The MolDock Score (GRID) scoring function was utilized with a grid resolution of 0.30 Å and a radius of 5 Å encompassing the entire binding cavity. The MolDock SE algorithm was employed for docking with 10 runs, a maximum of 1500 interactions, and a maximum population size of 50. The molecular docking simulation used a maximum evaluation of 300 steps with a neighbor distance factor of 1 and an energy threshold of 100.

### 3.9. Statistical Analysis

The multivariate analysis was conducted following the methodology described by [41] using Minitab 17^®^ software (free version, Minitab Inc., State College, PA, USA). The variables considered were the chemical constituents of the floral aroma. To ensure equal weighting, the raw data were first standardized. PCA was performed using the correlation matrix configuration provided by the software. For the Hierarchical Clustering Analysis (HCA) of the samples, the Euclidean distance measurement option and the complete connection method were employed. The multivariate analysis focused on chemical constituents that accounted for ≥0.5% of the total composition. Furthermore, a Pearson correlation study was conducted to analyze the relationship between classes of compounds and climatic data, aiming to assess their influence on the chemical composition.

## 4. Conclusions

The maximum yields of essential oil from *V. sebifera* leaves are achieved during the Amazonian winter (February) across all three specimens studied. The chemical profile of the essential oil from the leaves is predominantly characterized by sesquiterpene hydrocarbons, specifically (E,E)-α-farnesene. The chemical variation, both quantitative and qualitative, among the oils of *V. sebifera* specimens is found to be influenced by seasonality (Amazonian summer and winter), as well as the age of the specimens studied. In the experiment involving *A. salina* larvae, samples A and B, which had a high concentration of (E,E)-α-farnesene, exhibited high toxicity, while sample C demonstrated moderate toxicity. The greatest inhibition values in the DPPH tests were noted in samples from specimen B collected during the Amazonian winter, which had a lower concentration of (E,E)-α-farnesene. These findings enhance our understanding of the chemical composition of *Virola* species. As demonstrated, significant molecules have been identified in the EOs of *V. sebifera*, suggesting that this species can serve as a natural source of chemically active substances for a broad spectrum of industrial applications.

## Figures and Tables

**Table 1:** Moisture Content and Yield of EOs.

	Specimen A		Specimen B		Specimen C	
	September/2018	February/2019	September/2018	February/2019	September/2018	February/2019
Moisture (%)	9.44	10.72	10.5	11.39	8.95	10.70
Yield (%)	0.69	0.8	0.55	0.7	0.54	0.69

**Table 2:** Identified constituents in the EOs of three specimens of *V. sebifera* in September 2018 and February 2019.

IR_L_	IR_C_	Constituents	Specimen A		Specimen B		Specimen C	
			September/2018	February/2019	September/2018	February/2019	September/2018	February/2019
924	924	α-Thujene	0.16		0.18		0.17	0.24
932	932	α-pinene	6.93	3.15	11.95	5.03	6.37	16.91
969	971	Sabinene	0.47	0.22	0.34		0.4	1.2
974	976	β-Pinene	0.7		1.14		0.52	
981	984	6-Methyl-5-hepten-2-one		0.34				1.97
988	989	Myrcene	0.26		0.13		0.43	
1002	1005	α-Phellandrene	0.11					
1014	1016	α-Terpinene	0.1				0.07	
	1016	δ-2-Carene			0.1			
1025	1028	Sylvestrene	0.6		0.59		0.28	0.4
1044	1045	(E)-β-Ocimene	0.03				0.07	
1054	1057	γ-Terpinene	0.21		0.23		0.15	
1086	1088	Terpinolene	0.07		0.06		0.04	
1174	1177	Terpinen-4-ol	0.44	0.68	0.29	0.25	0.31	0.32
1186	1190	α-Terpineol	0.05				0.04	
1284	1286	Bornyl acetate	0.04	0.04	0.04		0.04	0.05
1324	1326	Myrtenyl acetate	0.05	0.05	0.09			0.12
1335	1342	δ-Elemene	3.56	3.27	1.94	4.3	4.34	1.47
1345	1352	α-Cubebene	0.14	0.17		0.1	0.12	
1374	1376	Isoledene	0.06		0.02			
1374	1379	α-Copaene	0.47	0.53		0.09	0.41	
1387	1389	ß-Bourbonene					0.13	
1389	1396	ß-Elemene	1.97	2.23	1.74	1.75	1.79	2.15
1417	1430	(E)-caryophyllene	12.26	16.02	19.67	19.34	15.7	21.4
1430	1436	ß-Copaene		0.44		0.51	0.75	
1434	1439	γ-elemene	2.7	2.92		6.81	7.01	
1439	1445	Aromadendrene	0.64	0.84	0.67	0.63	0.7	0.61
1442	1448	6.9-Guaiadiene	0.8	0.35		0.48	0.25	
1449	1451	Spirolepechinene		0.29				
1458	1456	Alloaromadendrene			0.11		0.86	
1452	1460	α-humulene	2.14	2.71	3.04	3.4	2.88	3.58
1460	1462	Dehydro aromadendrane		0.08	0.04			
1464	1466	9-epi-(E)-caryophyllene	0.54	0.64	0.43	0.66	0.64	0.58
1478	1478	γ-Muurolene		0.75		0.05		
1481	1479	γ-Himachalene			0.06			0.08
1484	1487	Germacrene D	3.14	2.81		7.33	7.72	
1489	1491	β-selinene	0.42	0.45	0.41			0.46
1483	1492	α-Amorphene				0.16		
1437	1494	α-Guaiene					0.38	
1495	1497	γ-Amorphene	0.32			3.55	0.59	
1500	1505	Bicyclogermacrene	5.69	8.85	5.6	7.29	5.63	5.5
1505	1522	(E.E)-α-Farnesene	47.57	42.82	47.65	23.57	26.65	37.43
1511	1528	δ-Amorphene				1.87		0.5
1522	1537	δ-Cadinene	1.08	1.2	0.29		1.68	
1529	1541	(E)-γ-Bisabolene	0.37	0.32	0.12			0.25
1533	1543	*trans-Cadina-1.4-diene*				0.06	0.19	
1537	1547	α-Cadinene	0.15					
1548	1556	Elemol	0.16	0.13		0.47	0.67	
1559	1566	Germacrene B	0.61	1.35		3.02	1.39	
1577	1584	Spathulenol	0.38	0.93	0.22	1	0.73	0.85
1592	1591	Viridiflorol	1.48	1.79	1.04	2.31	2.28	1.7
1600	1608	Rosifoliol	0.24	0.24	0.18	0.25	0.25	0.23
1608	1613	Humulene epoxide II		0.05				0.06
1618	1621	1.10-di-epi-Cubenol					0.08	
1632	1632	α-Acorenol				0.44		
1627	1634	1-epi-Cubenol	0.13				0.43	
1630	1638	γ-Eudesmol					0.28	
1639	1642	Alloaromadendrene epoxide		0.2	0.04	0.22		0.17
	1645	Naphth-1-ol			0.12			
1640	1647	epi-α-Muurolol	0.4			0.78	0.9	
1645	1649	Cubenol		0.06		0.15		
1644	1652	α-Muurolol					0.22	
1652	1656	Himachalol	0.11	0.58			0.32	
1651	1658	Pogostol						0.27
	1659	Selin-11-en-4α-ol			0.17			
1652	1660	α-Cadinol	0.48				1.06	
1671	1680	Tetradecanol					0.06	
1685	1691	Germacra-4(15).5.10(14)-trien-1-α-ol					0.04	
1700	1700	Eudesm-7(11)-en-4-ol	0.03			0.09		
1740	1744	Mint sulfide					0.04	
1755	1767	α-Sinensal					0.02	
1942	2114	Phytol					0.05	
Monoterpene hydrocarbons			9.64	3.37	14.72	4.58	8.5	18.75
Monoterpene oxygenated			0.49	0.68	0.29	0.25	0.35	0.32
Sesquiterpene hydrocarbons			84.63	89.04	81.79	84.97	79.81	74.01
Sesquiterpene oxygenated			3.41	3.98	1.65	5.71	7.28	3.28
Others			0.09	0.43	0.25	4.04	0.19	2.14
Total			98.26	97.5	98.7	95.96	96.13	98.5

**Table 3:** LC_50_ values of the three specimens of *V. sebifera* collected in September in a toxicity test against *Artemia salina*.

Especies	Concentrations (μg·mL^−1^)	Mortality (%)	R^2^	CL_50_ (μg·mL^−1^)
*V. sebifera* (Specimen A)	200	76.67	0.6037	70.92 ± 2.22
	150	46.67		
	100	46.67		
	50	40.00		
	25	36.67		
*V. sebifera* (Specimen B)	200	93.32	0.8619	72.17 ± 5.43
	150	63.33		
	100	60.00		
	50	40.00		
	25	16.67		
*V. sebifera* (Specimen C)	200	93.33	0.73	51.17 ± 3.95
	150	76,67		
	100	63.33		
	50	46.67		
	25	36.67		

## Data Availability

The data presented in this study are available on request from the corresponding author due to privacy.
